# Disease awareness may increase risk of suicide in young onset dementia: A case report

**DOI:** 10.1590/1980-57642016dn11-030015

**Published:** 2017

**Authors:** Maria Alice Tourinho Baptista, Raquel Luiza Santos, Nathália Kimura, Isabel Barbeito Lacerda, Marcia Cristina Nascimento Dourado

**Affiliations:** 1 MSc, Center for Alzheimer's disease and Related Disorders, Institute of Psychiatry, Universidade Federal do Rio de Janeiro, RJ, Brazil; 2 PhD, Center for Alzheimer's disease and Related Disorders, Institute of Psychiatry, Universidade Federal do Rio de Janeiro, RJ, Brazil

**Keywords:** young onset, dementia, Alzheimer disease, awareness, depression, suicide, início precoce, demência, doença de Alzheimer, consciência da doença, depressão, suicídio

## Abstract

Studies report that people with young onset Alzheimer's disease (YOAD) have higher levels of disease awareness compared to those with late onset AD. We report a case of a man with YOAD who had preserved awareness of disease, depression and risk of suicide associated with the development of the dementia. Cognitive functioning, disease severity, depressive symptoms and awareness of disease were assessed using validated measures. The person with YOAD showed a moderate level of disease severity and high degree of dependence for activities of daily living. There was recognition of memory problems and routine changes with presence of intense pessimism, low self-esteem and suicidal ideation. This case points to the existence of specific issues related to young onset dementia and the clinical importance of identifying and treating patients who might be aware of their condition.

## RESUMO

Estudos relatam que pessoas com doença de Alzheimer (DA) de início precoce possuem maior consciência da doença comparadas com as de início tardio. Descrevemos o caso de um homem com DA de início precoce, que apresenta consciência da doença preservada, depressão e risco de suicídio associado ao desenvolvimento da demência. O funcionamento cognitivo, gravidade da doença, sintomas depressivos e a consciência da doença foram acessados através de instrumentos validados. A pessoa com DA de início precoce apresentou um nível de gravidade da doença moderado e um elevado grau de dependência para as atividades de vida diária. Houve reconhecimento dos problemas de memória e mudanças de rotina com presença de pessimismo intenso, baixa autoestima e ideação suicida. Esse caso indica a existência de questões específicas relacionadas à demência de início precoce e a importância clínica de identificar e tratar pacientes que possam estar conscientes de sua condição.

## INTRODUCTION

The term Young-Onset Dementia defines all dementia-related conditions that commence before the age of 65.^1^ Alzheimer's disease is the most frequent cause of young-onset dementia.^2^^,^ 3 Young-onset Alzheimer's disease (YOAD) is characterized by faster decline and greater initial loss of cognitive abilities, particularly attention, visuospatial function and language.^4^

Disease awareness is the ability to recognize the impairments caused by a disease and involves three aspects: the ability to recognize a specific deficit;^5^^-^ 7 the emotional response to the difficulties; and the ability to understand the impact of the impairment on activities of daily living.^6^ Some studies have found an association between younger age of disease onset and deficit in awareness, but most have not specifically focused on YOAD.^5^^,^ 8 ^,^ 9

Van Vliet et al.^5^ found that people with YOAD have higher levels of awareness compared to those with late onset Alzheimer's disease (LOAD). In comparison to people with LOAD, individuals with YOAD have shown more depressive symptoms.^5^^,^ 7 In most YOAD cases, subjects may be depressed due to the awareness of their situation.^10^ In addition, there is evidence that people with mild dementia are at risk for suicidal behavior, often in the context of comorbid depression.^11^

The bulk of current research on awareness, depression and suicide risk focuses on LOAD. The current case report explores the experience of a person with YOAD, focusing on his preserved awareness of disease and the relationship with depression and suicide risk.

## METHODS

We report the case of 62-year-old man with YOAD attending an outpatient clinic for Alzheimer's disease in Rio de Janeiro, Brazil. Data were collected from the time of admission to the Clinic in 2011 up to 2016. Cognitive functioning, disease severity, depressive symptoms and awareness of disease were assessed with validated measures. Detailed anamnesis, clinical exams and previous cognitive measurements were taken from the patient's records. Written informed consent was obtained from both the patient and caregiver.

## CASE REPORT

The first symptoms occurred when the patient was 54 years old. The wife noticed memory loss regarding recent events and executive dysfunction (lost objects, difficulty in financial management, planning and organization of daily and professional tasks). They went to a neurologist and a psychiatrist and the diagnosis of AD was disclosed. Initially, he showed no reaction to the diagnosis, because he was worried with professional difficulties. Immediately after disclosure of the diagnosis, he stopped driving, working, walking alone, and a caregiver was hired. At this time, there were deficits in word recall and writing. One year later, he became aware of the cognitive deficits and presented depressive symptoms with suicidal ideation. He was "useless" in his own words. He reported sadness with his functional dependence and with the progressive nature of the disease. At the same time, he reported suicidal ideation. There was a spontaneously self-reported association between these symptoms and the disease awareness: "*You don't understand my suffering … I have Alzheimer, dementia…I will get worst and worst till I don't know anything anymore, not even who I am! It is better to die than stay this way*".

### Premorbid personality.

The patient was described as an extroverted, sensitive, supportive, but also an intransigent man. He was always anxious, considering the possibility of problems arising. He had outbursts of anger when upset, and was a perfectionist both with himself and his family.

### Imaging exams (done before arrival at the institution).

Cerebral Perfusion (2009) – moderate hypo-perfusion involving the posterior parietal lobes and the posterior portion of the cingulate gyrus. These findings suggested International Statistical Classification of Diseases and health related problems (ICD) 10 - F00.

### Cerebrospinal fluid examination (2010).

Determination of Biomarkers for Alzheimer's Disease: Beta Amyloid Protein (1-42) – 525 pg/mL (Reference: >540pg/mL); Tau protein 1036 pg/ml (Reference: <450 pg/ml); Phosphor-Tau 136 pg/ml Protein (Reference: <61 pg/ml).

### Measurements results.

The evolution of the cognitive and clinical deficits according to the progression of disease can be observed in the Mini-Mental State Examination (MMSE)^12^ and Verbal Fluency^13^ scores from 2011 up to 2015: MMSE (27 – 20 – 20 – 19 – 14) and Verbal Fluency (13 – 13 – 11 – 4 – 4).

In 2015, he scored 3 points, showing preserved awareness of the disease on the Assessment Scale of the Psychosocial Impact of the Diagnosis of Dementia ASPIDD.^14^ The patient recognized his memory problems, routine changes and reported being sadder than before due to health problems. His wife reported a moderate level of disease impairment^15^^,^ 16 (Clinical Dementia Rating = 2), with a high degree of dependence for ADL. The Pfeffer Functional Activities Questionnaire^17^ total score was 29 points, which indicates a very poor functional status. The Cornell Scale for Depression in Dementia score was 20.^18^^,^ 19 There was presence of intense mood and behavioral symptoms related to depression, intense anxiety, sadness, lack of reaction to pleasurable events, irritability, agitation, loss of interest in usual activities; intense suicidal ideation, accompanied by low self-esteem and pessimism. Total score on the Neuropsychiatric Inventory^20^^,^ 21 was 16 points with presence of delusions, irritability/labiality, disinhibition, agitation/aggression and apathy. The depressive symptoms were frequent and the wife reported that the patient had bouts of crying and regret, exhibiting sad and discouraged behavior. The wife reported that he depreciated himself, feeling that he had failed and had no future. Frequently, he verbalized being a burden to her, expressing desire and ideas of killing himself. According to her, his depression caused disturbance to his life, the symptoms were spontaneously verbalized by the patient, but it was difficult to attenuate them.

### Treatment.

He had been an outpatient of a centre for Alzheimer's disease since 2012. From 2013 to 2015, he attended a specialized day-care centre three times a week. The treatment plan included: music therapy; group psychotherapy to treat his mood labiality and promote acceptance and adaptation to his situation; cognitive stimulation, to enhance cognitive reserves and train compensatory strategies to maintain independence; and psychoeducational support group for caregivers. In 2016, he became aphasic, with severe executive dysfunction and was institutionalized in a nursing home.

Throughout the years of treatment, he was distressed when faced with his deficits. He usually became agitated and aggressive with his wife and the formal caregiver when they tried to help him. Sometimes he expressed a wish to die, sometimes he said he would kill himself. The clinical staff was concerned about his suicide ideation. The staff established interventions for the crises: individual meetings with the patient, formal caregiver and wife. Strategies of intervention were established with the wife to minimize environmental hazards, such as removal of sharp objects, protective nets on the windows, not leaving the patient alone, gripping his hands tightly when near roads. The staff tried to increase the family support by including the patient's sisters and children from the first marriage, but they refused to help.

By the end of 2015, he was taking Donepezil 5 mg, Bupropion 150 mg (one in the morning), Citalopram 20 mg (one in the morning), memantine 30 mg (one in the morning and one at night), Olanzapine 5 mg (one at night), Orcarbozepine 300 mg (one at night).

The sociodemographic and clinical characteristics are depicted in Table 1.

**Table 1:** Sociodemographic and clinical characteristics.

Gender	Male
Educational level	>8 years
Marital status	Married with children
Diagnosis	Alzheimer's disease
Age at onset	54
Age at diagnosis	55
CDR	2
MMSE	14/30
ADAS-Cog	43/70
Trail Making Test	A 185" percentile < 0.1% B after 300" failed to complete the task
Verbal fluency test	4
ASPIDD	3/30
QOL-AD	35
CSDD	29/30
PFAQ	20
NPI	16/144
ZBI	47

CDR: Clinical Dementia Rating; ADAS-Cog: Alzheimer Disease Assessment Scale – Cognitive Subscale; ASPIDD: Assessment Scale of Psychosocial Impact of the Diagnosis of Dementia; QOL-AD: Quality of Life in Alzheimer's Disease; CSDD: Cornell Scale for Depression in Dementia; PFAQ: Pfeffer Functional Activities Questionnaire; NPI: Neuropsychiatric Inventory; ZBI: Zarit Burden Interview.

## DISCUSSION

Studies have found that awareness decreases with disease severity.^22^^-^ 24 However, this case report showed a person with moderate YOAD and preserved awareness, despite decreased cognitive functioning and high level of dependence on activities of daily living.

Previous studies have reported differences in level of depression according to disease severity,^25^ e.g., depression may decrease in moderate YOAD. A possible explanation is related to the psychosocial changes among people with mild YOAD, and the difficulties coping with the familial and social adjustment, which may increase mood alterations.^25^ Conversely, our case report showed the presence of intense depressive symptoms. Beaufort and Vathorst^26^ found that people with Alzheimer's disease may have feelings of fear related to the disease progression. The distress associated with the future loss of autonomy, as well as becoming a burden upon relatives, may lead to feelings of hopelessness and suicidal ideation.^27^

Alzheimer's disease has been associated with a mild risk of suicide.^28^ However, the present case report is in line with research on the risk factors for suicide in dementia, which include depression, hopelessness, mild cognitive impairment, preserved insight, younger age, male gender, highly educated professional status, limitations in activities of daily living, economic stress, functional decline, and lack of social support.^11^^,^ 26 ^-^ 28

In this case report, it is plausible to assume that the pharmacological and non-pharmacological interventions may have had a significant effect on perceived health status, family and social support, depression, and coping, which may avoid a suicide attempt.^26^

Despite the limited generalizability of the evidence of the present findings, this case report emphasizes the need to further explore the associations between YOAD, awareness, depression and risk of suicide. This study highlights the clinical importance of evaluating disease awareness in people with YOAD and its link with risk of suicidal behavior, where clinicians should carry out an appropriate assessment of emotional needs when working with YOAD patients.
